# Incidence of malaria-related fever and morbidity due to *Plasmodium falciparum* among HIV1-infected pregnant women: a prospective cohort study in South Benin

**DOI:** 10.1186/1475-2875-13-255

**Published:** 2014-07-04

**Authors:** Alexandre Duvignaud, Lise Denoeud-Ndam, Jocelyn Akakpo, Komlan V Agossou, Aldric Afangnihoun, Didier G Komongui, Félix Atadokpédé, Lucien Dossou-Gbété, Pierre-Marie Girard, Djimon-Marcel Zannou, Michel Cot

**Affiliations:** 1 UMR 216, Institut de Recherche pour le Développement, Paris, France; 2 Université Paris Descartes, Paris, France; 3 Service de Médecine Tropicale, Clinical International Health, Centre Hospitalier Universitaire de Bordeaux, Bordeaux, France; 4 Centre de Traitement Ambulatoire, Centre National Hospitalier Universitaire Hubert Koutoukou Maga, Cotonou, Bénin; 5 Faculté des Sciences de la Santé, Université d’Abomey-Calavi, Cotonou, Benin; 6 Clinique Universitaire de Gynécologie et Obstétrique, Centre National Hospitalier Universitaire Hubert Koutoukou Maga, Cotonou, Bénin; 7 Centre de Traitement Ambulatoire, Hôpital de zone de Suru Léré, Cotonou, Benin; 8 Service de Gynécologie-Obstétrique, Hôpital de la Mère et de l’Enfant Lagune, Cotonou, Bénin; 9 Service de Médecine Interne, Hôpital d’Instruction des Armées, Cotonou, Bénin; 10 Clinique Louis Pasteur, Cotonou, Bénin; 11 Service des Maladies Infectieuses et Tropicales, Hôpital Saint-Antoine, Assistance Publique Hôpitaux de Paris, Paris, France; 12 Université Pierre et Marie Curie, Paris, France

**Keywords:** Malaria, Pregnancy, HIV, Fever, Incidence rate, Attributable fraction, Morbidity

## Abstract

**Background:**

Malaria and HIV are two major causes of morbidity and mortality among pregnant women in sub-Saharan Africa. Foetal and neonatal outcomes of this co-infection have been extensively studied. However, little is known about maternal morbidity due to clinical malaria in pregnancy, especially malaria-related fever, in the era of generalized access to antiretroviral therapy and anti-malarial preventive strategies.

**Methods:**

A cohort study was conducted in order to estimate the incidence rate and to determine the factors associated with malaria-related fever, as well as the maternal morbidity attributable to malaria in a high-transmission setting of South Benin among HIV-infected pregnant women. Four-hundred and thirty-two women who participated in a randomized trial testing strategies to prevent malaria in pregnancy were included and followed until delivery, with at least three scheduled visits during pregnancy. Confirmed malaria-related fever was defined as axillary temperature >37.5°C and a concomitant, positive, thick blood smear or rapid diagnostic test for *Plasmodium falciparum*. Suspected malaria-related fever was defined as an axillary temperature >37.5°C and the concomitant administration of an anti-malarial treatment in the absence of parasitological investigation.

**Results:**

Incidence rate for confirmed malaria-related fever was of 127.9 per 1,000 person-year (PY) (95% confidence interval (CI): 77.4-211.2). In multivariate analysis, CD4 lymphocytes (Relative Risk (RR) for a 50 cells/mm^3^ variation = 0.82; CI: 0.71-0.96), antiretroviral treatment started before inclusion (RR = 0.34; CI: 0.12-0.98) and history of symptomatic malaria in early pregnancy (RR = 7.10; CI: 2.35-22.49) were associated with the incidence of confirmed or suspected malaria-related fever. More than a half of participants with parasitaemia were symptomatic, with fever being the most common symptom. The crude fraction of febrile episodes attributable to malaria was estimated at 91%.

**Conclusions:**

This work highlights that malaria is responsible for a substantial morbidity in HIV-infected pregnant women, with cellular immunodepression as a major determinant, and establishes the possible advantage offered by the early initiation of antiretroviral treatment.

**Trial registration:**

PACOME Study has been registered under the number NCT00970879.

## Background

The consequences of malaria in pregnancy (MiP) for the foetus and the newborn have been extensively studied. MiP increases the risk of low birth weight (LBW), which is the most important determinant of mortality during the first year of life in African infants [1]. In mothers, MiP increases the risk of maternal anaemia [2-4]. Co-infection with HIV and *Plasmodium falciparum* has been associated with an increased risk of malaria-related fever among HIV-infected adults living in Uganda, especially in those with severe immunodepression [5,6]. The consequences of MiP are more severe [7-9], intermittent preventive treatment (IPTp) with sulphadoxine-pyrimethamine (SP) is less effective [10] and resistances to antifolates are more frequent [11] among HIV-infected than in HIV-uninfected women. Additionally, the risk of unfavourable outcomes related to MiP remains high in HIV-infected multigravid women [7,8,12]. Finally, the mother-to-child transmission of HIV could be facilitated [13]. One explanation for that could be the increase of HIV viral load during malaria-related fever episodes [14]. However, other studies found no [15,16] or an equivocal [17] association between placental malaria and mother to child transmission of HIV.

Despite this body of knowledge, data concerning the maternal morbidity attributable to *P. falciparum* infection in HIV-infected pregnant women, especially fever, are scarce. Incidence data on clinical malaria in this population are rare, ancient, and do not reflect the actual standard of care towards antiretroviral therapy access and anti-malarial preventive measures [18]. Moreover, this issue have never been studied longitudinally regarding potential associated factors [8,12].

To avoid the adverse consequences of MiP, the World Health Organization (WHO) now recommends IPT_p_ with a single dose of SP at each antenatal visit for all pregnant women, irrespectively of their HIV status [19]. However, due to the spread of resistances to antifolates in Africa [20-22], the evaluation of alternative strategies is urgently needed. The PACOME project was a randomized trial comparing daily cotrimoxazole to intermittent preventive treatment with mefloquine for the prevention of malaria in HIV-infected pregnant women living in Benin. It consisted of a prospective cohort of 432 HIV-1 infected pregnant women living in an urban-suburban area of South Benin [23]. This follow-up offered the opportunity to assess longitudinally malaria-related fever incidence rate and associated factors as well as the morbidity attributable to *P. falciparum* infection in this population.

## Methods

### Study site and participants

The study took place in the Littoral department of Benin, in Cotonou (the economical capital of Benin) and its suburbs, and in Porto Novo (the administrative capital), both located on the Atlantic coast of the Guinean Gulf, at the extreme south of the country. Regarding malaria, the south of Benin is characterized by a high perennial transmission of *P. falciparum* with two peaks during the rainy seasons, from April to June, and from September to November. The mean entomological inoculation rate was found to be 5.3 infective bites per person and per year in a close rural area [24] and the incidence of symptomatic malaria was 8.7% among adults older than 15 years living in the district of Cotonou in 2011 [25]. Among pregnant women, the nationwide prevalence of HIV infection was 1.6% in 2011. Of note, it was higher in the Littoral department (district of Cotonou), where it reached 3.4% [26].

HIV care centers and antenatal clinics (ANC) from five hospitals were involved in the study: Centre National Hospitalier Universitaire Hubert Koutoukou Maga, Hôpital d’Instruction des Armées de Cotonou, Hôpital de Zone de Suru-Léré, Hôpital Mère Enfant de la Lagune, and Clinique de Porto Novo. The main occupation of the women in this area is informal trading.

Midwives were recruited and trained as research assistants to fill questionnaires and to collect blood samples from the study participants. Local midwives, nurses and physicians in charge of the HIV care units and ANC worked in close collaboration with the research assistants.

### Study design

The study population consisted of 432 women enrolled in the PACOME clinical trial, between December 2009 and December 2011 [23]. The PACOME trial has been described in detail elsewhere [23]. Briefly, it was a randomized, non-blinded, non-inferiority trial (NCT00970879) aiming to compare the efficacy of two strategies for malaria prevention in HIV-infected pregnant women. The participants were randomized to receive either a daily cotrimoxazole prophylaxis throughout the course of pregnancy, or an IPTp with three intakes of mefloquine, between 16 and 28 weeks, 24 and 32 weeks, then 28 and 36 weeks of gestation. In case of low CD4 lymphocytes count (below 350 cells/mm^3^) or advanced HIV disease (WHO staging ≥2), all women received cotrimoxazole for the prevention of opportunistic infections, associated or not with mefloquine depending on the treatment group. Combined antiretroviral therapy (ART) was prescribed for the prevention of mother-to-child HIV transmission or for maternal health, according to national guidelines. The primary endpoint of the trial was the prevalence of placental malaria. All enrolled women had to be HIV1-infected and between 16 and 28 weeks of gestation, to live in the study area, to have planned to deliver at the hospital, to have signed an informed consent and to have no contra-indication to the study drugs.

According to the PACOME trial follow-up, women were evaluated at three antenatal care visits scheduled at least one month apart between 16 and 28, 24 and 32, 28 and 36 weeks, and at delivery. When sick, women were also encouraged to attend the clinic for free-of-charge diagnosis and treatment. Socio-economic data and obstetrical history were recorded at enrolment as well as HIV disease information. At each visit and at delivery, obstetrical, malaria and HIV-related information were also collected. In addition to anti-malarial preventive treatments, intravenous quinine was administered in case of severe malaria. A three-day treatment of artemether-lumefantrine (Coartem® Novartis) was administered in case of non-severe malaria, except for women with asymptomatic or low parasitaemia (<1,000 parasites/μl) who received a dose of mefloquine IPTp the same day. Opportunistic infections of HIV were treated according to national guidelines.

### Laboratory procedures

Laboratory procedures have been extensively described elsewhere [23]. Briefly, for malaria diagnosis, thick and thin blood smears were performed on each scheduled visit and at additional visits in case of symptoms suggestive of malaria. Blood smears were stained with Giemsa and microscopically examined for the detection of parasites. Malaria rapid diagnostic tests (RDTs) were also performed at each visit, scheduled or not, even in the absence of symptoms suggestive of malaria. The test used was Parascreen® (Rapid Test for Malaria Pan/Pf, Zephyr Biomedicals, Verna, Goa, India), a combined test detecting the histidine-rich protein 2 (HRP2) specific of *P. falciparum* and the *Plasmodium* lactate dehydrogenase (pan pLDH). HIV viral load was planned to be assessed at inclusion and delivery by real-time quantitative PCR using 2000 Abbott thermocyclers (Abbott Laboratories, Abbott Park, IL) provided by the National AIDS Control Programme, with a 40 copies per milliliter detection threshold. Absolute CD4 count and percentage were planned to be assessed at each visit by Cyflow (Partec, Münster, Germany) or Facscount (Beckton Dickinson, Franklin Lakes, NJ) cytometer.

### Definitions

Fever was defined as an axillary temperature >37.5°C. A case of confirmed malaria-related fever was defined as an axillary temperature >37.5°C and a concomitant, positive thick blood smear, or positive HRP2 band of the RDT in the absence of symptoms or findings indicating other infections. A case of suspected malaria-related fever was defined as an axillary temperature >37.5°C and the concomitant administration of anti-malarial therapy in the absence of parasitological investigation and of symptoms or findings indicating other infections.

### Statistical analysis

Data capture, cleaning and validation were performed using Microsoft Access 2003. Data analysis was performed using SAS version 9.2 for Windows (SAS Institute, Cary, NC, USA) and a two-sided 5% alpha risk.

First, women’s medical and socio-economic characteristics, the initial clinical presentation and, when known, the diagnoses associated with febrile episodes were described. The diagnoses associated with febrile episodes were compared according to the severity of cellular immunodepression. Incidence rates for confirmed malaria-related fever, suspected malaria-related fever and total malaria-related fever (confirmed or suspected) were estimated and reported symptoms associated with confirmed malaria were described. A sample size of 385 women was necessary to obtain an estimation of the incidence rate of malaria-related fever with 10% relative precision and a confidence level of 95% [27]. After univariate analysis comparing the characteristics of women with confirmed, suspected or without malaria-related fever (Fisher’s exact test for proportions and Wilcoxon’s exact test for quantitative variables), a multivariate Poisson regression was used to determine the association between women’s medical and socio-economic characteristics and the incidence of both outcomes (confirmed, then confirmed or suspected malaria-related fever). The initial models included the covariates with P-value <0.3 in univariate Poisson regression and those forced *a priori:* age, baseline CD4 cell count (as a quantitative variable assessed at inclusion), type of malaria chemoprophylaxis, study centre, parity, season at delivery. A stepwise procedure was used to select the strongest parsimonious models while searching for possible confusion or interaction between covariates. In the final multivariate analysis, a P-value <0.05 was considered significant. A sensitivity analysis was performed for the final model considering confirmed malaria-related fever as the dependent variable, first excluding suspected malaria fever cases from the analysis, then considering them in the control group.

Finally, the crude fraction of febrile episodes attributable to malaria among participants who were infected by *P. falciparum* from positive thick blood smear obtained on the occasion of scheduled visits was estimated. For this purpose, the following equation was used: AFe = (Re – R0)/Re, with AFe the attributable fraction of risk in exposed, Re the risk (of fever) in exposed and R0 the risk (of fever) in unexposed. Exposure was defined as having a positive blood smear for *P. falciparum* at the considered time point. The risk was defined as the number of either exposed or unexposed women having fever at the considered time point divided by the corresponding exposed or unexposed population (febrile and afebrile) at the same time point.

### Ethical considerations

The PACOME trial has been ethically approved both in France and in Benin, respectively by the ‘Comité consultatif de déontologie et d’éthique de l’IRD’, and by the ‘Comité national provisoire d’éthique pour la recherche en santé’, of the Beninese Faculté des Sciences de la Santé. The procedures followed were in accordance with the Helsinki Declaration of 1975, as revised in 2000.

## Results

### Baseline characteristics

Between December 2009 and December 2011, 432 participants were randomized in the PACOME study among 533 screened HIV-1 infected pregnant women. Of these, 140 received cotrimoxazole, irrespective of their treatment group, due to severe immunodepression or advanced HIV disease [23].

General characteristics of the participants at inclusion are summarized in Table 1. The mean age was 29.2 years (standard deviation (SD) 4.7 years) and mean gestational age was 21.7 weeks. Fifty-two per cent of women had a CD4 count below 350 cells/mm^3^, 52% had already initiated ART and HIV RNA viral load was undetectable in 82 (31.9%) women among 257 for whom this assay had been performed. Due to maintenance problems and financial shortages in National AIDS Control Programme laboratory, this test could not be performed for all women. For more details regarding ART regimen received by participating women see Additional file 1.

**Table 1:** Baseline characteristics of the 432 pregnant women who participated in the PACOME trial

**Category**	**Subcategory**	N (%) or mean (SD)*
**Sociodemographic characteristics**		
Health centre	CNHU	136 (31.5)
	HIA	76 (17.6)
	CLP	29 (6.7)
	HZ de Suru Léré	146 (33.8)
	HOMEL	45 (10.4)
Age (years) (N = 431)		*29.2 (4.7)*
Education	None or unknown	117 (27.1)
	Primary	153 (35.3)
	Secondary	128 (29.8)
	University	34 (7.8)
Marital status	Single	25 (5.8)
	Married (monogam)	272 (63.0)
	Married (polygam)	131 (30.2)
	Other or unknown	4 (1.0)
**General and obstetric characteristics**		
Obesity (BMI ≥30 kg/m^2^) (N = 427)		52 (12.2)
Sickle cell trait (N = 267)	Homozygote (SS)	16 (6.0)
	Heterozygote (AS)	26 (9.7)
Primigravid		50 (11.6)
Number of live born		*1.7 (1.5)*
Gestational age at inclusion (weeks)		*21.7 (3.8)*
Symptomatic malaria in early pregnancy before inclusion (N = 424)		45 (10.6)
**HIV–related characteristics**		
Time since HIV diagnosis (months)*		15 (1–42)*
WHO staging	1	303 (70.1)
	2	62 (14.4)
	3	65 (15.1)
	4	2 (0.5)
CD4 cell count (/mm^3^)*		342 (230–491)*
Undetectable viral load (N = 257)		82 (31.9)
Cotrimoxazole started before the ongoing pregnancy (N = 430)		204 (47.4)
Antiretroviral therapy started before inclusion (N = 429)		222 (51.8%)

*Values presented are median and interquartile range.

CNHU, Centre National Hospitalier Universitaire Hubert Koutoukou Maga; HIA, Hôpital d’Instruction des Armées de Cotonou; HOMEL, Hôpital Mère Enfant de la Lagune; CLP, Clinique Louis Pasteur; HZ, Hôpital de Zone.

### Follow-up

The 432 women who participated in the PACOME study totalized 130.9 person-years (PY) follow-up with a mean duration of 15.8 weeks (SD 5.6). Nine women (2.1%) were lost to follow-up just after their first visit and were not considered in incidence calculation. Recruitment and follow-up in the PACOME study have been detailed elsewhere. Briefly, 393 women (91%) were followed until delivery, with a median of 4 visits (interquartile range: 3 to 5). No death was imputed to the infection with *P. falciparum* among participants.

### Febrile episodes

Overall, 68 participants (16%) experienced a total of 86 febrile episodes, 17 of whom were associated with *P. falciparum* detection (positive HRP2 RDT and thick smear in 11 cases and isolated positive HRP2 RDT for six additional cases). Three febrile episodes were considered as suspected malaria by the caregivers and were treated by anti-malarial treatment. In total, 20 (23%) febrile episodes were referred as confirmed or suspected malaria-related fever. Six (30%) of these episodes, all related to confirmed malaria-related fever, occurred during the period between screening and inclusion of the participants, *i.e.* before the first intake of the trial’s preventive treatment. Clinical presentations of the other febrile episodes are summarized in Table 2. Eleven women experienced two febrile episodes (accounting for four confirmed malaria-related fever episodes, two of them occurring in the same woman, and one suspected malaria-related fever episodes), one woman experienced three febrile episodes (two of them being associated with *P. falciparum*) and one woman experienced six febrile episodes (no malaria-related fever).

**Table 2:** Clinical presentation of febrile episodes (n = 86) and diagnoses in the PACOME participants (N = 432)

**Clinical presentation - diagnosis**	**N (%)**
**Confirmed malaria**	17 (19.8)
Isolated positive RDT	6
Both RDT and thick blood smear positive	11
**Presumed malaria**	3 (3.5)
**Non-malaria fever**	66 (76.7)
Respiratory symptoms (including cough and dyspnea)*	5
Digestive symptoms (nauseas, vomiting, diarrhea) or pelvian discomfort	4
Cellulitis	1
Isolated fever	56

*one case of tuberculous pleuresia.

RDT, rapid diagnostic test.

### *Plasmodium falciparum* infections related morbidity

Forty-five non-recurrent episodes of confirmed infection with *P. falciparum* were reported during the PACOME trial. The symptoms reported by the women during these infections are summarized in Table 3. More than a half of them reported at least one symptom. Fever was the most frequently reported, present in one-third of cases. Headache, asthenia, nausea and vomiting were also reported. Fever was present in 46% (12/26) of the women infected with *P. falciparum* and having CD4 < 350/mm^3^ compared to 26% (5/19) of the women infected with *P. falciparum* and having CD4 ≥ 350/mm^3^ (p = 0.22, Fisher exact test).

**Table 3:** **Symptoms declared by the PACOME study participants with confirmed *Plasmodium falciparum *** infection* (n = 45)

	**N (%)**	
	**Positive RDT or TBS (n = 45)**	**Positive TBS (n = 32)**
Asymptomatic	21 (46.7)	14 (43.8)
Fever	17 (37.8)	11 (34.4)
Isolated fever	6 (13.3)	2 (6.25)
Headache	7 (15.6)	6 (18.8)
Asthenia	6 (13.3)	6 (18.8)
Nausea or vomiting	6 (13.3)	5 (15.6)
Muscular stiffness	3 (6.7)	3 (9.4)
Shivers	2 (4.4)	2 (6.3)
Cough	1 (2.2)	1 (3.1)

*assessed by rapid diagnostic test (RDT) or thick blood smear (TBS).

On the other hand, the proportion of febrile episodes referred as confirmed or suspected malaria-related fever was higher in women with a CD4 cell count <350/mm^3^ (31%) than in women with a CD4 cell count ≥350/mm^3^ (14%, p = 0.03). The distribution of febrile episodes and their suspected origin according to CD4 level are summarized in Table 4.

**Table 4:** Febrile episodes diagnoses (n = 86) in PACOME participants (N = 432) according to CD4 level

**Diagnosis**	**N (%)**		p-value*
	**CD4 < 350 by mm**^**3 **^**(n = 38)**	**CD4 ≥ 350 by mm**^**3 **^**(n = 30)**	
**Confirmed malaria**	12 (24.5)	5 (13.5)	0.17
Isolated positive RDT	5 (10.2)	1 (2.7)	
Both RDT and thick blood smear positive	7 (14.3)	4 (10.8)	
**Suspected malaria**	3 (6.1)	0 (0)	0.25
**Confirmed or suspected malaria**	15 (30.6)	5 (13.5)	0.03
**Total**	49	37	

*Fisher exact test.

Data regarding *P. falciparum* parasitaemia were available for 1,130 out of 1,626 scheduled visits. Thirty-one episodes of parasitaemia (non-recurrent positive thick smear) and 42 febrile episodes were recorded during scheduled visits. The distribution of the 1,130 visits according to the presence of parasitaemia or fever is summarized in Table 5. On this basis, the crude fraction of febrile episodes attributable to the infection with *P. falciparum* was 91%. In the 32 parasitaemic patients, the association between febrile condition and parasite density was investigated, when data were available. The median parasite density in febrile, parasitaemic patients was 1,000 trophozoïtes/mm^3^ (interquartile range (659–9,762), n = 10) *versus* 478 (interquartile range (157–1,422), n = 21) in afebrile, parasitaemic patients (p = 0.22, Wilcoxon exact test).

**Table 5:** **Distribution of scheduled visits according to the presence of *Plasmodium falciparum *** parasitaemia* and fever

		**Parasitaemia**		**Total**
		**Yes**	**No**	
Febrile episode	Yes	10	32	42
	No	21	1,067	1,088
Total		31	1,099	1,130

*assessed by systematic thick blood smear.

### Incidence rates estimations and associated factors

Unadjusted incidence rates for febrile episodes, confirmed malaria-related fever and suspected malaria-related fever are displayed in Table 6. The incidence rate of confirmed malaria-related fever was 127.9 for 1,000 PY (IC 95 = (77.4-211.2)) in the study population, which approximately corresponds to a proportion of 1/10 HIV-infected women undergoing malaria-related fever during the 40 weeks of gestation.

**Table 6:** **Incidence rates of febrile episodes and malaria-related fever for 1,000 PY (n = 432) (95**% **confidence interval)**

	
All febrile episodes	646.9 (497.8-840.7)
Confirmed malaria	127.9 (77.4-211.2)
Presumed malaria	22.6 (6.8-75.3)
Confirmed or presumed malaria	150.4 (94.9-238.5)

Concerning factors associated with incidence, in univariate analysis, baseline CD4 cell count at inclusion, considered as a dichotomic (threshold at 350 cells/mm^3^) or as a quantitative variable (p = 0.03) and having experienced symptomatic malaria (declarative data) at the beginning of the pregnancy (p = 0.001) were associated with confirmed or suspected malaria-related fever.

In the initial multivariate Poisson regression, modeling factors associated with confirmed or suspected malaria-related fever incidence, variables introduced apart from those forced *a priori* were the following: ART started before inclusion, complete follow-up until delivery, gestational age at inclusion and having experienced symptomatic malaria at the beginning of the pregnancy. In the final models (Table 7), confirmed malaria-related fever was associated to CD4 cell count at inclusion and having experienced symptomatic malaria at the beginning of the pregnancy. Confirmed or suspected malaria-related fever was associated with lower CD4 cell count at inclusion, having started antiretroviral therapy before the inclusion and having experienced symptomatic malaria at the beginning of the pregnancy. Of note, the type of antimalarial preventive treatment was not associated to malaria-related fever incidence. No interaction between the selected variables was evidenced. These results remained unchanged when performing sensitivity analysis (see Additional file 2).

**Table 7:** Factors associated with confirmed malaria fever incidence in the PACOME participants Multivariate Poisson regression*

**Variable**	**Outcome**			
	**Confirmed malaria (N = 410)**		**Confirmed or suspected malaria (N = 413)**	
	**Relative risk**	**95% CI**	**Relative risk**	**95% CI**
Age (for 5 years)	1.25	(0.70-2.27)	1.27	(0.74-2.18)
CD4 (for 50 cells/mm^3^)	0.83	(0.71-0.99)	0.82	(0.71-0.96)
Type of anti-malarial chemoprophylaxis (cotrimoxazole alone *vs* mefloquine IPT_p_ +/− cotrimoxazole)	1.72	(0.61-4.89)	2.24	(0.83-6.07)
Antiretroviral therapy started before inclusion	0.39	(0.12-1.21)	0.34	(0.12-0.98)
Symptomatic malaria during the early pregnancy (before inclusion)	8.89	(2.68-29.52)	7.1	(2.35-21.49)
Primiparity	1.09	(0.21-5.58)	0.84	(0.17-4.16)
Delivery during the rainy season	0.55	(0.20-1.54)	0.63	(0.25-1.60)

*adjusted on the health centre.

## Discussion

A study conducted in Rwanda during the early 1990s compared malaria incidence during the third trimester of pregnancy and six months post-partum among women infected or not by HIV [18]. The women were receiving neither antiretroviral therapy nor malaria preventive measures. For these reasons and due to definitions discrepancies, the overall incidence of clinical malaria infection was far higher than in the present study (6.2 per 100 women-months, *i.e.* 744 per 1,000 women-years, in the HIV-positive group and 3.5 per 100 women-months, *i.e.* 420 per 1,000 women-years, in the HIV-negative group, RR = 1.7, 95% CI = 1.4 – 2.3). Two other cross-sectional studies on malaria-related morbidity during pregnancy were carried out in Eastern Africa in the 1990s. They reported an increased frequency of anti-malarial drugs [8,12] use and of symptomatic malaria [12] in HIV seropositive compared to HIV seronegative pregnant women. In the first one, participants received IPT_p_ with two doses of SP, in accordance with local guidelines. In both studies, pregnant women had no access to ART. In the present work, almost all women were receiving ART and they all received anti-malarial chemoprophylaxis within the frame of the PACOME trial (IPT_p_ with mefloquine, daily cotrimoxazole or both). A noteworthy difference between the above-mentioned studies and this work is the earliness, the length and the quality of the follow-up. Of note, a similar analysis performed in a real life sample of HIV-infected pregnant women followed-up in the same centers, but not participating in the PACOME trial, provided comparable results for malaria-related fever incidence rates (Additional file 3). Therefore this study population is well representative of the population of HIV-infected pregnant women living in an urban area with a high transmission of *P. falciparum,* in the era of generalized ART, insecticide-treated bed nets use and IPT_p_.

The first important result is that among HIV-infected pregnant women receiving ART and adequate prevention of MiP, the incidence of malaria-related fever remained substantial. Although the malaria transmission conditions and population were not similar and the follow-up was much closer in the present study, one can compare the incidence rate of 127.9 per 1,000 PY for confirmed malaria-related fever found in this study’s population to the 101.6 per 1,000 PY found by French *et al.* in a population of HIV-infected adults with no access to ART or anti-malarial chemoprophylaxis living in Uganda [5]. Despite this, a residual under-reporting cannot be ruled out for some women with mild fever who might not have sought medical care.

This work confirms the role of CD4 immunodepression as a major determinant for the risk of malaria-related fever in HIV-infected pregnant women, already established in HIV-infected adults [5,6]. Furthermore, it suggests that febrile episodes could be more frequently related to malaria in the most immunocompromised women (*cf.* Table 4). However, this result has to be taken with caution, since it includes both confirmed and suspected malaria-related fever. An information bias related to a differential misclassification of suspected malaria-related fever episodes according to the severity of immunodepression could have occurred. Moreover, it is difficult to establish a causal relationship between febrile episodes and malaria in this population. In high transmission areas, an immunocompetent host can be infected with *P. falciparum* while experiencing fever or symptoms induced by other pathogens. In order to improve the specificity of malaria-related fever diagnosis, it has been proposed to take into account the parasite density in the definition of confirmed cases as well as for the estimation of the fraction of febrile episodes attributable to *P. falciparum* in parasitaemic subjects [28]. Among HIV-infected adults experiencing a febrile episode, it has been shown that the lower the CD4 cell count, the higher the parasite density [5]. Unfortunately, it was impossible to use such an approach in this study, because of the sparse available data on parasite densities. Moreover, when available, parasite densities were generally moderate or low and not very different according to the symptomatic status, probably because of preventive treatment.

No difference was found in malaria-related fever incidence according to the type of anti-malarial chemoprophylaxis. The high proportion of malaria-related fever episodes (30%) which occurred before the first intake of the trial’s preventive treatment is a possible explanation. However, a further analysis excluding these cases found similar results. The PACOME trial, whose primary endpoint was the placental infection by *P. falciparum* at delivery, concluded to the non-inferiority of daily cotrimoxazole compared to mefloquine IPT_p_ in the subtrial with mandatory cotrimoxazole due to advanced HIV disease. In the subtrial without mandatory cotrimoxazole, the target sample size was not reached so it was impossible to assess non-inferiority [23]. All of these results suggest that the levels of protection conferred by the different strategies used in this population are probably not very different.

Having experienced symptomatic malaria in early pregnancy appeared to be strongly associated with the incidence of malaria-related fever during the follow-up in multivariate analysis. The hypothesis was made that this variable reflects the role of unmeasured factors, for instance, an overall higher exposure to malaria transmission in symptomatic pregnant women. Delivering during the rainy season did not constitute a risk factor for malaria-related fever in this work. The majority of the participants were living in an urban area, less favourable to malaria transmission than a rural setting. Additionally, the time of delivery may not be an adequate indicator of the exposure to malaria associated with seasonal variations throughout the whole pregnancy.

In the multivariate analysis of factors associated with confirmed and suspected malaria-related fever episodes, ART initiated before inclusion appeared to be a protective factor, even after adjusting for baseline CD4 count. This could be due to an important gain in CD4 during follow-up for women under ART for a short time before inclusion. The only CD4 cell count included in the analysis was performed at baseline because of frequent missing data for further assessments, so it is not possible to further elucidate this question. This finding represents an additional reason to start ART as early as possible during the pregnancy especially in women newly diagnosed of their HIV infection. Another study carried out previously in the same cohort confirmed the benefit of early ART during pregnancy in order to reach an undetectable HIV viral load at delivery and to minimize the risk of mother-to-child transmission of the virus [29]. The control of HIV replication could also play an important part in the observed association between longer ART duration and decreased malaria fever incidence.

An earlier study in Uganda had already shown that ART associated to daily cotrimoxazole was protective against malaria-related fever, compared to daily cotrimoxazole alone, in a population of HIV-infected adults and adjusted for the CD4 cell count [30]. In the Ugandan study, as in the present study, the time since seroconversion and the CD4 slope were not taken into account. These variables could improve the assessment of HIV infection severity and thus the possibility to evaluate the benefit of early ART intake on the risk of symptomatic malaria.

MiP in HIV-negative pregnant women living in a high-transmission area is generally considered to be asymptomatic [31]. However, previous studies suggested that MiP is frequently symptomatic and responsible for a high morbidity in HIV-infected pregnant women, especially with low CD4 [5,9]. Overall, more than one third of the PACOME participants who were infected by *P. falciparum* on a systematically performed blood smear were febrile at the same time. Interestingly, the frequency of fever among women infected with *P. falciparum* was almost doubled when the CD4 count was low, although not significantly. The efficacy of malaria preventive measures and a possible lack of power are possible explanations. It could be valuable to systematically record malaria-related maternal morbidity in all longitudinal studies aiming to assess new strategies for the prevention of MiP, especially in vulnerable populations, such as HIV-infected pregnant women. From this point of view, studies including both HIV-infected and uninfected pregnant women are highly suitable to allow direct comparison.

## Conclusions

In Benin, malaria-related fever is responsible for a substantial morbidity among HIV-infected pregnant women in spite of generalized access to ART and adequate anti-malarial prevention. More than a quarter of febrile episodes were related to malaria in the study population. This justifies prompt and accurate diagnosis and treatment of malaria within the frame of antenatal care programmes. The severity of immunodepression, which has already been shown to be major risk factor for malaria-related morbidity in HIV-infected adults, is of special concern among HIV-infected pregnant women. As a consequence, women with low CD4 cell count should be prioritized regarding access to ART and anti-malarial chemoprophylaxis.

Indeed, the earliness of ART during the pregnancy could be an efficient measure for the prevention of malaria-related morbidity in this population. From a pathophysiological point of view, the hypothetic restoration of a parity-dependent phenomenon for the acquisition of anti-malarial protective immunity under ART should be further assessed. It could be a strong argument for immediate and definitive ART upon diagnosis of HIV infection for all pregnant women (option B + of the WHO) [32]. It also raises the problem of global implementation of ‘test and treat’ strategies in resource-limited settings. Finally, women who have already presented a malaria-related fever episode during their pregnancy should be followed more closely because they are likely to present re-infections with *P. falciparum*.

## Competing interests

The authors declare they have no competing interest.

## Authors’ contributions

AD and LDN carried out the acquisition, the statistical analysis and the interpretation of data and drafted the manuscript. MC participated in the design and the coordination of the study, the acquisition of data, supervised the writing and revised critically the manuscript. JA, KVA, AA, DGK, FA, LDG, and DMZ participated in the acquisition of data. PMG and DMZ participated in the design of the study and revised critically the manuscript. All authors read and approved the final manuscript.

## Supplementary Material

Additional file 1 Antiretroviral regimen received by PACOME trial participants. Click here for file

Additional file 2 Factors associated with confirmed malaria-related fever episodes incidence in the PACOME trial participants: sensitivity analysis including the suspected malaria-related fever episodes (n = 3) in the non malaria fever group. Click here for file

Additional file 3 Incidence rates of febrile episodes, confirmed and presumed malaria-related fever episodes for 1000 PY in a real life sample of 174 HIV-infected pregnant-women followed-up in 4 out of 5 PACOME study sites. Click here for file
